# Behavioral, Biochemical and Electrophysiological Changes in Spared Nerve Injury Model of Neuropathic Pain

**DOI:** 10.3390/ijms21093396

**Published:** 2020-05-11

**Authors:** Francesca Guida, Danilo De Gregorio, Enza Palazzo, Flavia Ricciardi, Serena Boccella, Carmela Belardo, Monica Iannotta, Rosmara Infantino, Federica Formato, Ida Marabese, Livio Luongo, Vito de Novellis, Sabatino Maione

**Affiliations:** 1 Department of Experimental Medicine, Division of Pharmacology, University of Campania Naples, 80138 Naples, Italy; enza.palazzo@unicampania.it (E.P.); flaviaricciardi93@gmail.com (F.R.); boccellaserena@gmail.com (S.B.); belardocarmela85@gmail.com (C.B.); monica.iannotta@gmail.com (M.I.); rosmainfantino@gmail.com (R.I.); francesca.guida@unicampania.it (F.F.); ida.marabese@unicampania.it (I.M.); livio.luongo@gmail.com (L.L.); vito.denovellis@unicampania.it (V.d.N.); 2 Neurobiological Psychiatry Unit, Department of Psychiatry, McGill University, Montréal, QC H3A1A1, Canada; danilo.degregorio@mail.mcgill.ca

**Keywords:** spared nerve injury, neuropathic pain, behavior, electrophysiology, immune cells

## Abstract

Neuropathic pain is a pathological condition induced by a lesion or disease affecting the somatosensory system, with symptoms like allodynia and hyperalgesia. It has a multifaceted pathogenesis as it implicates several molecular signaling pathways involving peripheral and central nervous systems. Affective and cognitive dysfunctions have been reported as comorbidities of neuropathic pain states, supporting the notion that pain and mood disorders share some common pathogenetic mechanisms. The understanding of these pathophysiological mechanisms requires the development of animal models mimicking, as far as possible, clinical neuropathic pain symptoms. Among them, the Spared Nerve Injury (SNI) model has been largely characterized in terms of behavioral and functional alterations. This model is associated with changes in neuronal firing activity at spinal and supraspinal levels, and induces late neuropsychiatric disorders (such as anxious-like and depressive-like behaviors, and cognitive impairments) comparable to an advanced phase of neuropathy. The goal of this review is to summarize current findings in preclinical research, employing the SNI model as a tool for identifying pathophysiological mechanisms of neuropathic pain and testing pharmacological agent.

## 1. Introduction

The ability to experience pain possesses a protective role since it warns of imminent or ongoing tissue damage and elicits behavioral responses in order to minimize this damage. Whether tissue harm is inevitable, a plethora of excitability changes in the peripheral or the central nervous system (PNS and CNS, respectively) establish a profound, but reversible pain hypersensitivity in the inflamed tissue. This process assists injury repair because any contact with the damaged part is avoided until healing has occurred. However, chronic pain states, including neuropathic pain, offer no biological advantage and cause suffering and distress [1]. Neuropathic pain can be triggered by injuries affecting the PNS or CNS and is characterized by spontaneous or abnormal pain sensation [2]. The source of neuropathic pain development cannot be always established or reversed, and the available pharmacological tools are unsatisfactory. Pathophysiology of neuropathic pain is complex. Imbalances between excitatory and inhibitory somatosensory signaling, alterations in ion channels and abnormal immune reactions associated with enhanced plasticity have been implicated in neuropathic pain states. The discovery of new targets and the development of novel pharmacological approaches need a variety of preclinical animal models, whilst translating preclinical observations to new, targeted drug therapies in patients, which is a major challenge. Thus, the understanding of the underlying pathophysiological mechanisms requires the development of preclinical models mimicking, as far as possible, clinical neuropathic pain symptoms in humans. Different models have been conceived in order to reproduce disease-like conditions such as diabetic neuropathy, chemotherapy-induced neuropathic pain, antiretroviral drugs induced neuropathy and spinal or peripheral nerve damages. Surgical approaches, such as the peripheral nerve ligation or transection [3,4,5,6,7], represent well-validated models to reproduce neuropathic pain following PNS damage in rodents. Injury to the sciatic nerve is usually used because it is easy to access and is relatively large size. Both peripheral and central effects are observed including spontaneous discharge from afferent neurons, nociceptor sensitization, and spinal and supraspinal reorganizations. Such models differ in location or procedure of the injury by reproducing different aspects of neuropathic pain syndromes in humans (for review please see [8]), but their exhaustive presentation is beyond the scope of this review. Here, we focus on the Spared Nerve Injury (SNI) model, developed by Decostered and Woolf [9], which relied on the axotomy of two of the three branches of the sciatic nerve. This model presents several technical advantages and induces long-lasting behavioral and functional dysfunctions (see Box 1) [8,10,11,12,13].

The SNI model has been largely characterized in term of both sensory (nociceptive) and emotional (affective) dimensions. It has proven to induce a prolonged increase in mechanical sensitivity and thermal (hot and cold) responsiveness ipsilaterally to the injury. In addition to pain sensory symptoms, the neuro-psychological functioning is affected by SNI, and the complex forebrain network is considered the substrate for negative affective states and cognitive impairments [9,14,15]. Behavioral modifications are associated with a maladaptive reorganization of the nervous system due to the changes in neurotransmitter release and neuronal firing activity at spinal and supraspinal levels in which neuron-glia cells communication takes place [16]. Indeed, it is accepted that SNI-mediated pain development is triggered by the activation of glia cells, microglia (early) and astrocytes (late), contributing to the neuronal sensitization at spinal cord level. The contribution of peripheral immune cells and possible infiltration of T cells in the spinal cord after SNI has been also suggested [17]. Glia activation-driven neuroinflammation has been widely described in the brain. Indeed, changes in neural plasticity in affective and somatosensory regions, induced by SNI, may play a crucial role in the onset and development of negative affective components of pain such as anxiety, anger and depression. Interestingly, recent evidence indicates a relationship between gut microbiota and pain, suggesting a role for “microbiota-gut-brain axis” in the development of chronic pain [18,19]. Contribution of gut microbiota in SNI-mediated sensorial modifications or depression in mice has been described [18,20].

This review contains recent preclinical findings that pertain to the employment of the spared nerve injury as model of neuropathic pain in rodents. Specific modifications in terms of behavior, biochemistry and electrophysiological activity in SNI animals have been reported.

Box 1 **Pros and cons of spared nerve injury model.**

**Advantages:**

- The surgical procedure is relatively easier than other methods and offers high reproducibility
- SNI permits behavioural testing of the non-injured sural nerve territory (adjacent to the denervated areas).
- As unilateral injury, the SNI-induced behavioural signs or biochemical markers can be compared with to contralateral side.
- SNI does not affect daily life activity, such as food intake, drinking and locomotion or circadian patterns [12]. Autonomy is not observed, unlike other models (i.e. Sciatic Nerve Transection).
- As compared with the chronic constriction injury (CCI), SNI induces more intense and prolonged (up to one year) mechanical sensitivity [10,12]. The long-lasting pain behaviours allows, similarly to the clinical situations, to perform chronic treatments (several weeks or months) after onset of symptoms and clear diagnosis (therapeutic treatment).

**Disadvantages:**

- SNI produces a low local inflammation that is present in the CCI or partial sciatic nerve ligation (PSNL) and SNL models [8].
- Apart from recent developed scales to assess facial expression of pain in rodents [11,13], behavioral signs of spontaneous pain are difficult to be measured.
- Sciatic nerve injuries in humans are rare to due the deep anatomical location within the lower extremity.

## 2. Surgical Procedure

Fully anaesthetized rodents are placed on a warmed pad and the surgical site is shaven by using a chemical depilatory cream or an electrical razor to minimize contaminations. An incision (1 cm for mouse or 1.5 cm for rat) is performed in the longitudinal direction proximal to the knee by using a surgical scalpel. After the exposure of the sciatic nerve through the biceps femoris muscle, the common peroneal and the tibial nerves are tightly-ligated (nonabsorbable 6.0 silk for mouse or 3.0 silk for rat) and sectioned distal to the ligation, removing 2–4 mm of the distal nerve stump. The sural nerve is left intact. Using sutures or clasps, the muscular layer and the incision in the shaved layer of the skin are stitched. Sham controls involve the exposure of the sciatic nerve and its branches without any lesion. The lesion results in evident hypersensitivity in the lateral area of the paw, which is innervated by the spared sural nerve [21].

## 3. Behavioral Symptoms Associated with the Spared Nerve Injury

The SNI produces sensory symptoms such as mechanical and thermal hypersensitivity to noxious (hyperalgesia) and innocuous (allodynia) stimuli, which develop immediately after 3 days and last between 4 weeks [22] to 12 months in mice [10] and to 7 months in rats [23] following the injury. Intriguingly, even the sham-operated controls show an initial mechanical hypersensitivity immediately after surgery that returns to baseline thresholds within days, an event which is probably related to the inflammatory state produced by the surgery [22]. Variants of the SNI model that have injured the tibial nerve alone produced mechanical hypersensitivity, whereas those that have injured the common peroneal and sural nerves together did not increase the mechanical threshold in mice tested with von Frey filaments [24]. The same outcomes have been described for the same SNI variants in rats [25]. Rats subjected to SNI exhibit hyper-responsiveness to supra-threshold thermal stimuli in the plantar test and cold allodynia in the acetone test [9]. Interestingly, the sensory symptoms that occur after SNI, such as mechanical allodynia and hyperalgesia, hyper-responsiveness to suprathreshold thermal stimuli and cold allodynia coincide with the sensory anomalies reported by patients suffering from neuropathic pain [1,26]. All the behavioral tests used for assessing severity and progression of pain measures the latency to withdraw or escape from a thermal or mechanical stimulus: a higher latency equates to a higher nociceptive threshold. These tests (as those for evaluating anxiety, depression-like behavior, and cognitive impairment) depend on the motor activity of the animals, which remains unaffected in the SNI model of neuropathic pain [27,28]. However, the animal tests for the measurement of pain responses that are evoked by stimuli are difficult to translate to the human condition in which spontaneous pain is the most disabling symptom. It has also to be taken into consideration the fact that the spontaneous pain is under-studied in animal models. In the last decade, the group of study led by Mogil et al. developed a mouse grimace scale (MGS), a standardized behavioral coding system in order to assess, with high accuracy and reliability, noxious stimuli of moderate duration accompanied by facial expressions of pain [11]. Noteworthy, facial pain expression represent a big opportunity for the understanding of the physiology and for the management of neuropathic pain. For instance, it has been highlighted that facial expression have been found similar in both human and nonhuman animals [29] and, therefore, the understanding of the sensorial and neurophysiological perturbances induced by the multifaceted neuropathic pain condition could benefits from a deeper investigation of facial expression of rodents following SNI surgery.

Apart from sensory abnormalities, chronic pain impairs mood and cognitive processes, thereby, worsening the quality of life in humans [30]. Chronic pain is among the main causes of affective disorders. The same comorbidity among chronic pain and affective disorders is found in animal models of chronic pain [31,32]. Accordingly, anxiety and depression-like behaviors have been widely reported in the SNI model of neuropathic pain [28,33,34,35,36,37,38,39]. Anxiety-like behavior following SNI has been found in both rats and mice. In rats, anxiety-like behavior has been observed in the elevated plus-maze and open field tests at different postoperative times: day 14 [40], day 21 [40,41], day 28 [34,42], days 20–40 [43] and it continues for between 8 weeks [44] and 24 weeks [38]. Interestingly, other studies have failed to observe anxiety-like behavior in the rats subjected to SNI using the same tests over a long observation period ranging from 7 to 19 weeks [27,45]. There is just as much evidence that the SNI model of neuropathic pain is associated with the development of anxiety-like behavior in mice. In particular, anxiety-like behavior has been observed in the elevated plus-maze and open fields tests at day 14 [37,46], day 23 [47], day 28 [48] and weeks 4–6 [49,50] after injury. Anxiety-like behavior was also observed in the black box emergency test on day 12 [35], in the marble-burying test, which also measures compulsive behavior [51] at day 30 [36,37], in the light-dark box on day 30 [36] and in the hole board test in weeks 4–6 [49]. In the open field, no anxiety-like behavior was observed on day 7 [28], from day 3 to week 7 [12] and from day 3 to week 14 [52] after the SNI. Urban et al. [12] also found a lack of anxiety-like behavior in the elevated zero-maze and marble-burying while Pitzer et al. [52] in the elevated plus-maze and hole board. It is certainly not easy to understand the reason for this inconsistency. The time factor is certainly critical [53,54] since measuring anxio-depressive behavior at very early or late time can produce misleading outcomes. Other factors (and a combination thereof) that can generate variability in behavioral responses are the species, the strain, the night-day cycle in which the animals are tested, and the tests used.

The SNI model of neuropathic pain also induces depression-like behavior in rodents (including anhedonia and despair-like behavior). In rats, for example, the development of depression-like behavior was observed in the sucrose preference and forced swimming tests on day 14 [39,55,56], day 18 [56], day 21 [57], day 25 [58], day 28 [34], day 42 [59,60], day 45 [43], day 49 [27], day 56 [39,59] and week 11 [61] from the surgical insult. Surprisingly, only one study failed to detect depression-like behavior in the sucrose preference test 3 weeks after SNI surgery in the rat [62]. Similarly, in the mouse, the SNI induces depression-like behavior in the forced swimming, sucrose preference, and tail suspension tests at different times from the surgery: from day 3 to week 7 [12], day 7 [28,63], day 14/15 [37], day 30 [33,36], week 6 [50], week 9 [46] up to one year [10]. Similar to rats, a single study found no depression-like behavior in the forced swimming test from the 3rd to 97th day following the SNI surgery in mice. Interestingly, this same study did not even detect anxiety-like behaviors under the same time point and pain conditions [52].

Pain affects cognition in humans and rodents since it can divert attention, memory, and decision-making [64]. However, the amount of studies investigating the cognitive deficits associated with the SNI model of neuropathic pain is less than those that have focused on affective disorders [10,33,36,65,66,67,68]. This discrepancy could reflect the complexity, number, and diversity of paradigms available for measuring cognition. Most studies on cognitive performance in rats undergoing SNI have focused on working memory, an ability to retain spatial information for a short time. Working memory deficits were detected in rats mainly using mazes, devices, which exploits the innate leaning (and remembering) of the location associated with safety, food or any other reward in rodents [68]. Working memory impairments were detected at 3–7 days [65] and persisted for a month [34] after SNI surgery. Additionally in rats, attention and reaction time were reduced after 1 month and 3–6 months after the SNI [42,69,70]. Curiously, these attention deficits were subject to laterality: cognitive impairment was observed mainly when SNI was performed in rats on the right side [42]. Recognition memory in the novel object recognition task was also impaired in rats subjected to SNI at day 7 [71], days 12–15 [72], day 21 [71] until day 45 after surgery [73]. In mice, most studies investigated recognition memory in the novel object recognition, finding deficits on day 14 [37], days 28–30 [36,74] months 4th [67] and months 12th from SNI surgery. Deficits in spatial memory were also found in SNI mice on day 14 [37,75] and 30 [36] from surgery. Impairment in cognitive flexibility was also detected in mice in the attentional rule-shifting task 21 days after SNI surgery [76]. Curiously, no impairment in spatial working memory was found in the Morris water maze 3 to 21 days after SNI in mice by Karl et al. [77]. All the evidence described so far highlights close comorbidity existing between neuropathic pain in general and the SNI model in particular and disturbances of affectivity and cognition. A schematic summary of behavioral effects of SNI model is given in the Table 1.

Similar to rodents, in humans there is also a close correlation between neuropathic pain and cognitive/affective disorders [79,80]. This is due to the fact that chronic pain causes anatomical and functional alterations in the main neural circuits that control both, pain and cognition/affect [79]. This overlap also explains why reciprocally psychological procedures such as distraction, yoga and meditation can be effective in some individuals and pain conditions and how the expectation of pain-relief is associated with placebo-induced analgesia in humans [80].

## 4. Role of Glia and Immune Cells in the SNI Model

In SNI model of neuropathic pain is nowadays accepted that there is the participation of non-neuronal cells of the CNS (astrocytes, microglia). Meanwhile, recent findings indicate the cooperation of central and peripheral immune cells is crucial for the development of the tactile allodynia. Indeed, in young animals that do not have a mature immune system, tactile allodynia and the clear microglia activation, are not evident and do not develop until rats are 4 weeks of age at the time of the SNI induction [81].

Microglia and astrocytes are responsible for the neuronal wellbeing by exerting a defense mechanism in pathological conditions, including neuropathic pain [82,83]. Several studies have presented evidence for the role of persistent microglia (early) and astrocytes (late) activation (indicated by changed morphology or overexpression of specific surface antigens) in the spinal cord in the induction and maintenance of allodynia after SNI [84,85,86]. In addition, different neuro-immune interactions have also been proposed. Under activation, both microglia and astrocytes show an hypertrophic phenotype and release several cytokines [i.e., interleukin-1β (IL-1β), IL-6 and tumor necrosis factor α (TNF-α)] that able to bind their specific receptors on neuronal surface by triggering neuronal sensitization and contributing to the inflammatory reaction. Indeed, intrathecal injection of minocycline (specific microglia inhibitor) led to the reversal of SNI-induced allodynia [78]. Activation and proliferation occur in the ipsilateral dorsal horn of the spinal cord together with an overexpression of the phosphorylation of the p38-mitogen-activated protein kinase (p-p38-MAPK) which consistently, represents an activation marker of the microglia [87]. Activation of p38MAPK in spinal cord has been associated with the downregulation of the glucocorticoid receptors (GRs) expression and activation NF-κB, which in turn induces the release of IL-6 and TNF-α [88].

The protein levels of P2X purinoceptors 4 (P2 × 4Rs) and brain-derived neurotrophic factor (BDNF) are upregulated in the spinal microglia of SNI animals by contributing to the allodynia development [84,89]. Chemokines are also strongly affected by the neuropathic pain condition. In particular, SNI increases the expression of C-X-C motif chemokine 12 (CXCL12) and its cognate receptor CXCR4 in dorsal root ganglia (DRG) neurons and satellite glial cells. SNI also induces long-lasting upregulation of CXCL12 and CXCR4 in the ipsilateral L4–5 spinal cord dorsal horn, characterized by CXCL12 expression in neurons and microglia, and CXCR4 expression in neurons and astrocytes [90]. Increased expression levels of the chemokine prokineticin (PK)2 have been observed in the astrocytes, but not microglia, in 7-days SNI mice [91]. Kanda et al., [92] have showed that microglia-derived TNFα induces cyclooxygenase 2 (COX2) and prostaglandin I2 (PGI2) synthase expression in spinal endothelial cells, suggest a glia-endothelial cell interaction in SNI condition. Kiyoyuki et al., [93] have suggested that leukotriene B4 from microglia and its cognate receptor leukotriene B4 receptor 1 (BLT1) expressed on spinal dorsal horn neurons, both increased in SNI.

Supraspinal CNS regions show changes in microglia and astrocytes phenotypes. Gui et al. [72]. detected reactive microglia in the prefrontal cortex, nucleus accumbens, amygdala and CA1 region of the hippocampus at day 12 after SNI. Here, microglia-derived IL-1β overproduction has been correlated with memory deficit and depression following peripheral nerve injury. Indeed, not only spinal but also brain inflammation, especially at chronic stages, may offer a clue to the mechanisms of analgesic, anxiolytic and antidepressants drugs in reducing neuropathic pain. The intensifying of microglial density was also observed in the infralimbic cortex of 7 day-SNI animals by Chu et al. [94]. Increased levels of pro-inflammatory molecules including TNFα, nitric oxide synthase (iNOS), Caspase-1 and IL-1β release have been detected in microglia or astrocytes in the medial prefrontal cortex of SNI animals [78,94,95]. Compared with controls, glial activation, coupled with increase of glial transporters (GTs), has also presented in the amygdala of 3-day SNI rats [96]. SNI induced microglia activation associated with BDNF reduction in hippocampus [73,97]. BDNF levels are also reduced in the prefrontal cortex of rats [57] and mice [33] with depression-like phenotype. Gosselin et al., [98] showed a robust microglia and astrocytes activation together with an up-regulation of GABA transporter GAT-1 in the gracile nucleus ipsilaterally to the site of injury in rats 7 days post-injury.

**Figure 1:**
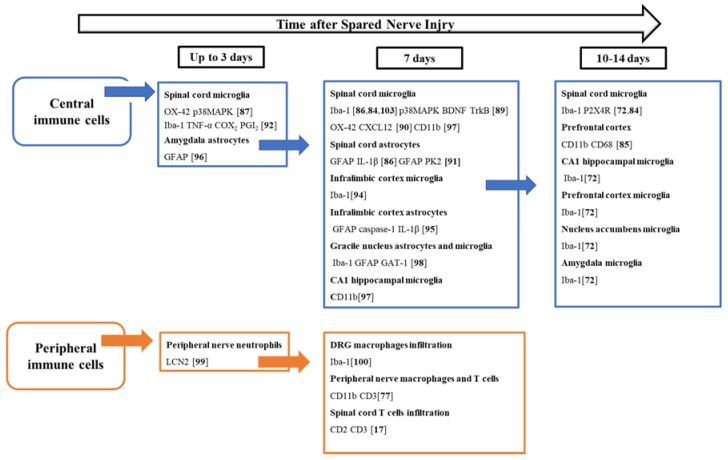
Schematic flow-diagram showing the temporal participation of central and peripheral immune cells after spared nerve injury. The scheme represents key time-points of immune cells activation, which occur throughout the nervous system, particularly at the site of nerve injury, in the dorsal root ganglion (DRG), the spinal cord, and supraspinally, within the brain. Mediators or antigens surface shown are expressed by the cell types indicated. Ionized calcium binding adaptor molecule 1 (Iba-1); Glial Fibrillary Acidic Protein (GFAP); cluster of differentiation molecule 11B (CD11b); cluster of differentiation 68 (CD68) cluster of differentiation 3 (CD3); cluster of differentiation 2 (CD2); CD11b/c Monoclonal Antibody (OX-42); P2X purinoceptor 4 (P2X4R); P38 mitogen-activated protein kinases (p38MAPK); Brain-derived neurotrophic factor (BDNF); Tropomyosin receptor kinase B (TrkB); Lipocalin-2 (LCN2); interleukin 1β (IL-1β); CXCL12 (C-X-C Motif Chemokine Ligand 12); Prokineticin-2 (PK2); GABA transporter type 1 (GAT-1); Tumor necrosis factor alpha (TNF-α); cyclooxygenase-2 (COX-2); Prostacyclin (PGI2) [17,72,77,84,85,86,87,89,90,91,92,94,95,96,97,98,99,100,103].

Recent findings suggest the contribution of peripheral immune cells in SNI-induced pain processing. Neutrophils are commonly the first peripheral immune cells to invade sites of injury. In SNI, increased neutrophils-derived Lipocalin 2 (LCN2) expression was found in damaged sciatic nerves 1 day after injury, suggesting its involvement in the early inflammatory response in the peripheral tissues [99]. Significant infiltration of macrophages was observed by Vega-Avelaira et al. [100] in SNI animals 7 days post injury. Their observations showed high levels of transcriptional activity in dorsal root ganglia (DRG) following nerve injury in adulthood compared to nerve injury in early life, sustained the reduced incidence of neuropathic pain in infants. Macrophages and lymphocytes invasion into the peripheral nerve has been observed 1 week after SNI [77]. The contribution of T cells in SNI-induced pain has been reported by Costigan and coll. (2009) [17] where they highlighted that mechanical allodynia is prevented in T-cell-deficient recombination activating gene 1 (Rag1)-null adult mice following SNI. Similar findings have been obtained using Rag2^−/−^ mice, which did not develop mechanical pain hypersensitivity after SNI surgery. The authors confirmed that Rag2^−/−^ mice reconstituted with T cells behave like WT mice in response to SNI [101]. Interestingly, lymphocyte infiltration into the spinal cord after SNI is still being debated. Different T-cell populations have been showed to infiltrate into the dorsal horn of spinal cord in 7-days SNI mice by Costigan et al. [17]. However, more recent evidence suggests that there is not a relevant infiltration of T cells in the SNI rats, although T-cell-derived cytokines were found to have increased and be possibly responsible of the activation of microglia [102]. Interestingly, the absence of spinal infiltration of T cells and mechanical hyperalgesia has been demonstrated in neonatal rats (less than P21) compared to adults, this coincided with expression of interferon gamma (IFN-γ), a critical pro-inflammatory mediator released by T helper 1(Th1) cells [17], whereas T cells and microglia with predominantly Th2 and M2 profile were described in neonates. A schematic representation of chronological participation of central and peripheral immune cells has been given in the Figure 1 [17,72,77,84,85,86,87,89,90,91,92,94,95,96,97,98,99,100,103].

Therefore, the crosstalk between central and peripheral immune cells could not be excluded in the SNI-mediated neuropathic pain, even if further investigations are needed to better define the cellular substrates and their communications in the establishment of the symptoms (pain and comorbidities) associated with the SNI. 

## 5. Sex Differences in SNI-Associated Pain Signaling

Evidence suggests that neuroimmune modulation of pain may be responsible for sex differences in pain behavioral outcomes. Although males and females display similar development of allodynia associated with SNI [104], recent findings suggest a gender-dependent immune-driven induction of pain. The focal point for any discussion of neuroinflammation is microglia. After peripheral nerve injury, microglia activation in the spinal cord progresses through a hypertrophic morphology, proliferation, and changes in gene expression. It has been suggested that in both males and females there is a marked proliferation of microglia after SNI. Interestingly, Brings and Zylka [105] revealed that SNI induces the overexpression of spinal microglial P2X4 receptors in male animals, but not in females. Thus, even if microglia assume an activated phenotype following SNI in both sexes, they might not be crucial in facilitating neuropathic pain behavior in females [106,107]. Accordingly, intrathecal injection of glia inhibitors such as minocycline, fluorocitrate or propentofylline, reduced the tactile allodynia in SNI males, but not in the females [103]. In their study, Sorge et al. reported that microglia are not required for mechanical pain hypersensitivity in female mice, while they achieved similar levels of pain hypersensitivity using adaptive immune cells, likely T cells. These data suggest a complex interaction between T cells and sex in pain signaling, although the physiology of these interactions remains unexplored. Interestingly, a more recent study showed that metformin, a prescribed drug for type II diabetes, had a disease modifying effect on male mice, which included a decrease in microglial activation in the spinal cord when given to reverse or to prevent neuropathic pain. Interestingly, metformin did not appear to have any observable effects on neuropathic pain or microglial activation in female mice [108]. While the evidence in preclinical research supports the hypothesis of a sex-dependent establishment of pain, thereby opening up a new scenario that was previously neglected, the specific etiological basis underlying these differences is unknown.

## 6. Electrophysiological Characterization of SNI Model

Electrophysiology is employed in order to investigate fundamental mechanisms contributing to the neuropathic pain state. Animals subjected to SNI surgery show a neuronal reorganization, (so-called neuroplasticity), characterized by dramatic changes in neuronal firing activity at both spinal and supraspinal levels. Nerve injury enhances presynaptic excitatory input onto spinal superficial dorsal horn neurons, by inducing changes in local postsynaptic circuits that lead to exacerbated behavioral responses [109]. In fact, changes also reach remote regions including the brainstem, limbic system and cortex. This reorganization in turn would be reflected on spinal cord processes through descending modulatory pathways and would also affect pain-related cognitive abilities and mood disorders [54].

In vitro and in vivo electrophysiology reveals an overall neuronal hyperexcitability at spinal cord levels after SNI, associated with increased glutamate signaling [110,111]. By performing, single-unit extracellular recordings, it has been found that the C-fiber response of dorsal horn Wide Dynamic Range (WDR) neurons (located in the lamina V) increased significantly in SNI rats compared with that in sham rats [112]. An enhancement of spontaneous and mechanical-evoked activity in the Nociceptive Specific (NS) neurons (located in the lamina II), associated with increased spinal expression of the pronociceptive prokineticin type 2, has been recorded 7 days post injury [91]. The enhanced neuronal excitation in the spinal cord induced by SNI can return to normal levels, concurrently with the resolution of the pain state [33,109]

The prefrontal cortex is strongly implicated in pain processing and in the integration of emotionally salient information related to a chronic pain condition [113]. Different studies demonstrated that the SNI model induces changes in medial prefrontal cortex (mPFC) neuron electrical activity associated with an increase in the extracellular glutamate levels, as measured by micro-dialysis [95,114]. Intriguingly, Guida et al. [33] observed that 30 days-SNI mice showed an overall decreased activity of mPFC pyramidal neurons with a subsequent reduced level of glutamate correlated with pain-related depression-like behavior, cognitive impairments, and obsessive-compulsive-like disorder. Sang et al. [44] suggested that the mPFC endures plastic changes after SNI (15 days), as indicated by an elevation of serotonin transporter (SERT) expression, as well as, a greater enhancement of its local field potential (LFP) theta-frequency associated with anxiety-like behavior in avoiding anxiogenic environment. The anxiety-like behaviors of the SNI rats were effectively suppressed by local application of serotonin to the mPFC.

SNI-induced pain facilitation involves also the recruitment of the pain-responding neuron populations in the Rostral Ventromedial Medulla (RVM). In this area, Fields and colleagues [115,116] identified two main different populations of RVM neurons, named ON and OFF cells, which receive neuronal inputs from the periaqueductal grey (PAG) and project to the dorsal horns in the spinal cord, an axis (PAG-RVM-spinal cord) known as descending nociceptive pathway. Just before the occurrence of reflexes induced by noxious stimuli, the firing rate of ON cells displays a dramatic increase (or burst firing) of the activity, and these neurons play a pro-nociceptive role. In contrast, OFF cells are characterized by a cessation (or pause) of the firing activity, and their activation produces antinociception. Palazzo et al. [117] showed that SNI procedure causes an increase of the ongoing and the pinch-related burst activity of the ON cells and a decrease of the OFF cells activity, with an increase of the pause duration.

A number of findings indicated neural activity changes in response to SNI in brainstem areas, including dorsal raphe nucleus (DRN) [47] the ventral tegmental area (VTA) [118,119] and the rostromedial tegmental nucleus (RMTg) [120], as well as, amygdala [114] and other limbic areas involved in chronic pain, as a possible substrate for the emotional-affective comorbidities. After 14 days, a discharge rate of serotonin DRN neurons and burst firing of VTA dopaminergic (DA) cells were enhanced in SNI rats, when compared with sham-operated animals. Increased DA neuron activity was paralleled by a decreased inhibition evoked by electrical stimulation of the RMTg, one of the major inhibitory inputs to DA cells [120]. In a recent study, Boccella et al. [74] showed an altered spatial memory and object recognition associated with impaired long-term potentiation (LTP) at the lateral entorhinal cortex-dentate gyrus (LEC-DG) pathway in 30 days-SNI mice. Indeed, unlike controls, SNI mice showed the complete absence of LTP following theta-burst stimulation (TBS), preceded by a higher basal fEPSPs amplitude and slope in response to a single pulse. Neuropathy also decreased postsynaptic density, volume and dendrite arborization of DG and induced substantial modification in glutamate levels and glutamate metabotropic receptors expression. Instead, the level of the endocannabinoid 2-arachidonoylglycerol (2-AG) was increased in the LEC.

## 7. Cutting-Edge Techniques in SNI Model: Focus on Optogenetic and DREADD

The study of neuropathic pain processes with the use of the pharmacological stimulation remains a valid approach, although it shows some deficits such as the lack of specificity when targeting a specific brain region or a specific neurotransmission. This represent a big limitation in the study of the neuronal/neural circuitry involved in the pathophysiology of neuropathic pain [121]. For instance, spatially specifically targeted strategies to modulate and to study the physiology of neuropathic pain have been developed and they could ultimately represent such a good tool to help science and clinical pain management. In the last decade, striking techniques such as optogenetic and Designer Receptors Exclusively Activated by Designer Drug (DREADD) have allowed many researchers to improve the knowledge in the field of neuroscience.

Optogenetic: optogenetic approach allows specifically regulate neuronal activity in neural circuits by providing insights into the mechanisms underlying pain processing. The main concept is that a light-sensitive ion channel is expressed in targeted cells, allowing for neuronal depolarization or hyperpolarization with pulses of light [122]. In most of cases, this requires the intra-cerebral infusion of viral vectors expressing a promoter for a specific neuronal population. After several weeks post injection, the expression of light-sensitive proteins selectively inhibit or activate the neuronal activity with the use of LASER or LED stimulation, thus producing behavioral effects [122,123,124]. More specifically, channel-rhodopsins are cation channels able to depolarize of neurons when illuminated. In other cases, neuronal activity can be shut down through illumination of the chloride pump halo-rhodopsin or the proton pump archeo-rhodopsins, both have the ability to hyperpolarize neurons. Since these proteins can be selectively expressed in specific cell types and in specific brain regions, the optogenetic approach avoids issues such as non-specific outcomes of electrical or pharmacological brain stimulation. In some cases, transgenic animals can be created with opsin expression under the control of general or specific promoters. For optogenetics in other species that are less tractable for transgenic models, such as rats, or for potential human therapeutic use, viral transduction of cells provides the best route for opsin expression [125]. In this case, a surgical procedure for intracranial implant is necessary to deliver the light pulses, but surely, the advantage of optogenetics is the ability to have precise temporal control of neuronal activity.

Several groups deeply investigate the application of this tool to modulate peripheral neural circuits ultimately opening new paths and landscape for the investigation of neuropathic pain and its neuronal/neural circuit [121,126,127,128,129]. In the context of the SNI model, a growing body of literature strongly contributed to a better understanding of chronic pain processes. For instance, it has been demonstrated that, mPFC local networks or functional connectivity between the mPFC and other brain regions display abnormal activities to normally innocuous stimuli, when such stimuli induce tactile allodynia, under SNI condition [130]. Optogenetic stimulation of the pyramidal neurons (following channel-rhodopsins expression) in the PFC has been shown to produce strong antinociceptive effects by reducing mechanical allodynia and depressive symptoms of pain in SNI model in rats [131]. This study also revealed that the projection from the mPFC to the nucleus accumbens is involved in the anti-nociceptive effects induced by optogenetic activation of the neuronal circuit. Similarly, the inhibition of prefrontal projections to the nucleus accumbens (NA) enhanced pain sensitivity in SNI rats, as shown by Zhou et al., [132]. Moreover, a recent study indicated that photoinhibition of excitatory glutamatergic neurons of mPFC improved the performance level (working memory) and restored neural activity to a similar profile observed in the control animals [66]. Likewise, Zang et al. [133] showed that activation of inhibitory archaerhodopsin or excitatory channel-rhodopsinin pyramidal cells in the prelimbic area of the PFC decreased and increased pain responses, respectively, and modulated the avoidance behavior in SNI animals.

Interestingly, Huang et al. [119] identified the basolateral amygdala (BLA)–prefrontal cortex (PFC)–periaqueductal gray (PAG)–spinal cord pathway as crucial circuit in SNI pathophysiology. Indeed, specific optogenetic inhibition of BLA inputs into the mPFC reversed mechanical allodynia and thermal hyperalgesia and affective components of pain, through a reduction of descending noradrenergic (NE) and serotonergic (5-HT) modulation of spinal pain signals [119,134]. Moreover, an impressive study demonstrated the contribution of input from the mediodorsal thalamus (MD) to the anterior cingulate cortex (ACC). In the study, the authors demonstrated that the optogenetic activation of MD inputs (following channel-rhodopsin expression) strongly exacerbated the mechanical hypersensitivity in SNI mice [129]. In addition, using a novel transgenic mouse in which the terminals of peripheral nociceptors were silenced with high degree of spatiotemporal precision, a recent study demonstrated that a prolonged optical silencing of peripheral afferents in anesthetized genetic mutant mice expressing archaerhodopsins on Nav1.8 channels dramatically decreased mechanical and thermal hypersensitivity under SNI conditions [135]. In addition, other researchers generated adult mice selectively expressing the outward rectifying proton pump archaerhodopsin in peripheral neurons expressing calcitonin gene-related peptide-α (CANs), and inhibited their peripheral cutaneous terminals in models of SNI with a transdermal light system activation. After SNI surgery, the authors found that brief activation of archeo-rhodopsin on their peripheral cutaneous terminals reversed chronic mechanical, cold, and heat hypersensitivity [136]. In addition, the role of optogenetic stimulations of glia cells has been as well investigated. More specifically, an outstanding study carried out by the group of Nam et al. showed that, following channel-rhodopsin expression, the photo-stimulation of astrocytes in the spinal cord exacerbated the mechanical allodynia in rats after 3, 7 and 10 days post SNI [137]. However, within the experimental techniques, several limitations occur. For example, the first limitation to take into consideration is that the level of stimulation risks to drive abnormal neuronal responses outside the physiological range, something that is particularly hard to assess because it is not usual practice to record neural activity simultaneously with behavioral experiments and this can lead to unnatural plasticity in the neural/neuronal circuit. Another limitation is characterized by the fact that the light stimulation and optogene expression are not uniform across the target neuron population; thus, generating heterogeneity in spatial extent of optogenetic manipulation or, in worst case, brain damage due to the heat that the light induces (for details, please refer to these articles [123,125].

DREADDs: in the context of chemogenetic, this approach has been defined as a method by which proteins are engineered in order to interact with previously unrecognized small molecules. Of these various classes of chemogenetically-engineered proteins, the most widely used to date have been the DREADDs. Those are used ubiquitously to modulate G protein-coupled receptors (GPCRs) activity in vivo, and have been widely applied in the field of behavioral neuroscience [138]. In particular, DREADDs that are activated by the inactive clozapine-N-oxide (CNO) have been emerged as the most adopted technology [139]. In pain, DREADDs have been used to understand specific neuron activity in the brain that control pain responding and output, and for the identification of new therapeutic targets. Compared with optogenetic, DREADDs are ideally suited for prolonged modulation of cell activity in the range of minutes or hours having implications for the study of behavior [140]. An outstanding study investigated the contribution of the somatostatin (SOM) interneuron firing activity to the hyperactivity of cortical L5 pyramidal neurons under neuropathic pain condition. To do so, the authors specifically infected SOM cells of somatosensory cortex (S1) with a viral vector encoding a specific DREADD receptor in a mutant line of SNI mice. The intraperitoneal administration of the CNO activated these receptors, thus promoting the Gq-coupled signaling activation. This induced a membrane depolarization via inhibition of potassium voltage-gated (KCNQ) channels, which ultimately increased the neuronal firing of SOM cells and reduced the activity of cortical L5 pyramidal neurons. This effect was coupled with reduced pain sensation in SNI mice [141]. Another work attempted to characterize the role of the infralimbic (IL) cortex during an approach-avoidance task. This task was characterized by the disruption of approach due to a pain-predictive cue (PPC-avoidance), extinguished by experience and reinstated in the SNI model. In particular, following the expression of selective DREADDs receptors, the chemogenetic inactivation of pyramidal neurons reinstated pain-predictive cue (PPC)-avoidance in SNI mice [142]. Furthermore, DREADDs application strongly contributed to corroborate the implication of the anterior nucleus of paraventricular thalamus (PVA) in the development of mechanical hyperalgesia due to the SNI model [130]. In particular, the study showed that direct inhibition of the PVA neuronal activity using the Gi-coupled DREADDs completely blunted chronic mechanical hyperalgesia in mice following SNI surgery. Recently, Pan and colleagues highlighted the role of subpopulation of excitatory interneurons (Ins) expressing Urocortin 3::Cre (Ucn3+) in the dorsal spinal cord as a central node in the pathway that modulate mechanical itch sensitization under neuropathic condition. In detail, the silencing of spinal Ucn3+Ins produced an attenuation light punctate stimuli (0.7 mN)-evoked mechanical itch in SNI mice [143]. As optogenetic, also DREADDs technique owns limitations. First, we have to take into consideration that neuronal manipulation requires a temporal control on the orders of milliseconds. While this is possible using optical methods, the neuronal modulation with DREADDs requires a temporal control of minutes in the activation or silencing of neurons. Therefore, CNO can take up to 2 h to be cleared from plasma [144], thus delaying the ability to halt neuronal modulation. A further hurdle arises when DREADDs are employed to investigate therapeutic targets. In case of over expression, DREADDs can easily over exceed the physiological levels of endogenous receptors. This overexpression can bring one to incorrectly target endogenous GPCRs in specific cell types in an attempt to mimic these results, therefore leading to beguilingly implicate these targets for pharmacological intervention. A solution to solve this issue can be the quantification of DREADD expression levels and the comparison to the levels of endogenous receptors. Indeed, this could provide more clarification whether the physiological outcomes will translate when endogenous receptors are directly targeted, as suggested elsewhere [13].

Overall, optogenetic and DREADDs are undeniably unveiling the role of the neurocircuitries involved in the pathophysiology of neuropathic pain, although the knowledge of many neurophysiological processes still remains elusive. Therefore, more research needs to be performed. Many questions concerning the potential therapeutic use of these tools in treating neuropathic pain, especially in clinical trials, have not yet been answered and deserve more investigation and attention from the neuro-scientific community.

## 8. Conclusions

Animal models of neuropathic pain are commonly employed in order to screen new compounds for their analgesic activity, as well as to investigate possible novel pathways in pain pathophysiology. However, the poor correlation between experimental symptoms and clinical signs, or the lack of reliable techniques to study spontaneous pain, may represent major limitations [145].

**Figure 2:**
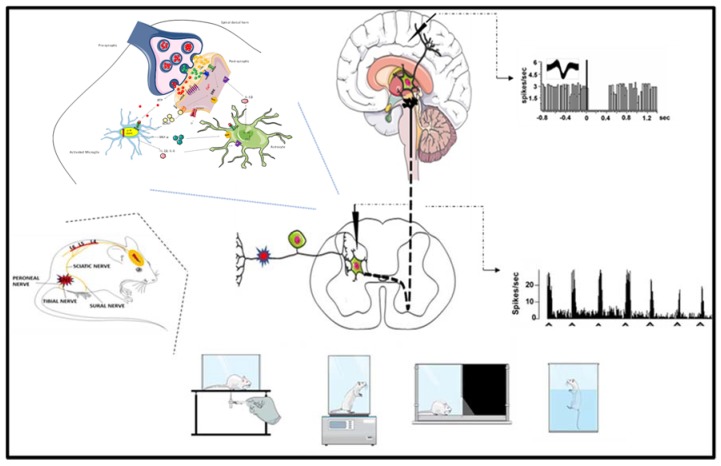
Summary scheme showing behavioral, biochemical and electrophysiological changes occurring after spared nerve injury (SNI).

Among them, the spared nerve injury has proved to be a robust model of neuropathic pain; thus, demonstrating substantial and prolonged changes in behavioral measures of mechanical sensitivity, thermal responsiveness, and related negative affective and cognitive consequences. Moreover, the spared nerve injury model induces altered glial homeostasis, neuronal excitability, synaptic plasticity and transmission in spinal and supraspinal areas altogether defined as the ‘pain matrix’ (Figure 2). The model closely mimics the fundamental alterations and symptoms of clinically described neuropathic pain disorders, which is fundamental for the study of pathophysiological mechanisms.

## Figures and Tables

**Table 1:** Behavioral effects of SNI model.

Surgery	Specie and Sex	Effect	Duration	Methods	References
SNI	Male rat	Mechanical allodynia and hyperalgesia Thermal hyperalgesia	from 24 h to 7 months	Von Frey Pin prick Acetone/Ethyl Cloride test Hargreaves’ test	[10,24]
Crush injury of tibial and common peroneal nerves			from 4 days to 7 weeks		
Spared common peroneal	Male rats	Mechanical and cold allodynia	From day 4 to 10 weeks		[25]
Spared tibial		Mechanical allodynia No cold allodynia	only at 14 days		
Spared common peroneal and sural		Mechanical and cold allodynia			
Common peroneal, tibial and sural nerves injured		Mechanical and cold allodynia			
SNI	Male and female mice	Mechanical Allodynia	From 3 to 28 days	Von Frey	[24]
	Male mice		Until 12 months	Dynamic Plantar Aesthesiometer	[10]
Spared tibial nerve	Male mouse	Mechanical allodynia	From 3 to 14 days	Von Frey	[7]
Spared sural and common peroneal	Male and female mice	No mechanical allodynia Mechanical allodynia	From 3 to 28 days	Von Frey	[7,24]
SNI	Male rats	Anxiety-like behavior	14 days	Light dark box	[40]
		Anxiety-like behavior	21 days	Open field, elevated plus maze	[41,62]
		Anxiety-like behavior	23 days	Open field, elevated plus maze	[47]
		Anxiety-like behavior	28 days	Open field, elevated plus maze	[34,42]
		Anxiety-like behavior	20–40 days	Open field, elevated plus maze	[43]
		Anxiety-like behavior	4–8 weeks	Open field, elevated plus maze	[44]
		Anxiety-like behavior	24 weeks	Elevated plus maze	[38]
	Female rats	No anxiety-like behaviour	8 weeks	Open field, elevated plus maze	[27]
		No anxiety-like behaviour	From 2 to 19 weeks	Open field, elevated plus maze	[45]
SNI	Male mice	Anxiety-like behavior	12 days	Fear condition and extinction, black box emergency	[35]
	Male mice	Anxiety-like behavior	14 days	Open field, elevated plus maze, marble burying	[37,46]
		Anxiety-like behavior	28 days	Open field, elevated plus maze	[48]
	Male mice	Anxiety-like behavior	30 days	Light dark box, Marble burying	[36]
	Male and female mice	Anxiety-like behavior	4–7 weeks	Elevated plus maze, light dark box, holeboard	[49]
	Male mice	Anxiety-like behavior	6 weeks	Open field, elevated plus maze	[50]
	Male mice	No anxiety-like behavior	From day 3 to week 7	Elevated zero maze, marble, burying	[12]
	Male and female mice	No anxiety-like behavior	From 3 to 97 days	Elevated plus maze, hole-board	[52]
	Male rats	Depression-like behavior	14 days	Forced swim, sucrose preference	[55,78]
	Male rats	Depression-like behavior	13–16 and 20–23 days	Forced swim, sucrose preference	[57]
	Male rats	Depression-like behavior	14 and 18 days	Forced swim, sucrose reference	[56]
	Male rats	Depression-like behavior	14 and 56 days	Forced swim, sucrose preference	[39]
	Male rats	Depression-like behavior	day 25	Forced swim, sucrose preference, tail suspension	[58]
	Male rats	Depression-like behavior	day 28	Forced swim	[34]
	Male rats	Depression-like behavior	12 and 19 day	Sucrose preference	[60]
	Male rats	Depression-like behavior	42, 56 days	Forced swim, sucrose preference	[59]
	Male rats	Depression-like behavior	42 days	Sucrose preference	[43]
	Male rats	Depression-like behavior	7 weeks	Forced swim	[27]
	Male rats	Depression-like behavior	11 weeks	Sucrose preference	[61]
	Male rats	No depression-like behavior	3 weeks	Sucrose preference	[62]
SNI	Male mice	Depression-like behavior	3 days to 7 weeks	Forced swim, sucrose preference	[12]
	Male mice	Depression-like behavior	7 days	Forced swim	[28,63]
	Male mice	Depression-like behavior	14 days	Tail suspension	[37]
	Male mice	Depression-like behavior	30 days	Forced swim, Tail suspension	[33,36]
	Male mice	Depression-like behavior	6 weeks	Tai suspension, sucrose preference	[50]
	Male mice	Depression-like behavior	9 weeks	Forced swim	[46]
	Male mice	Depression-like behavior	1 year	Tail suspension	[10]
	Male mice	No depression-like behavior	3 to 97 days	Forced swim	[52]
